# The Lifeworld of the Complex Care Hospital Doctor: A Complex Adaptive Phenomenological Study

**DOI:** 10.1007/s10728-023-00474-8

**Published:** 2024-01-19

**Authors:** Felice Borghmans, Stella Laletas, Harvey Newnham, Venesser Fernandes

**Affiliations:** 1 Faculty of Education, Monash University, Melbourne, Australia; 2 School of Educational Psychology and Counselling, Melbourne, Australia; 3 Central Clinical School, Monash University, Melbourne, Australia; 4 Alfred Health, Melbourne, Australia

**Keywords:** Lifeworld, Complex, Phenomenology, Chronic, Multimorbid, Healthcare, Hospital

## Abstract

The ever-increasing prevalence of chronic conditions over the last half century has gradually altered the demographic of patients admitted to acute care settings; environments traditionally associated with episodic care rather than chronic and complex healthcare. In consequence, the lifeworld of the hospital medical doctor often entails healthcare for a complex, multi-morbid, patient cohort. This paper examines the experience of providing complex healthcare in the pressurised and fast-paced acute care setting. Four medical doctors from two metropolitan health services were interviewed and their data were analysed using a combinatorial framework of phenomenology and complexity theory. The horizon of complex care revealed itself as dynamic, expansive, immersive, and relational, entailing a specialised kind of practice that is now common in acute care settings. Yet this practice has made inroads largely without heralding the unique nature and potential of its ground. Herein lies opportunity for complex care clinicians to expand notions of health and illness, and to shape research, practice, and system design, for a future in which care for health complexity is optimised, irrespective of care settings.

## Background

Since the mid-last century, the prevalence of chronic and complex conditions has seen a perpetual increase that has paralleled population health measures and advances in medical science [1, 2]. In consequence, the term ‘epidemic’ now describes the global prevalence of chronic conditions, and it is equally applicable to the Australian context [3]. Almost half of the Australian population has one chronic condition, while 20% have two or more chronic diseases [4, 5]. It follows that chronic conditions were involved in 52% of hospitalisations in 2019–20 [5]. It is also widely acknowledged that hospital care in its current form is a reactive and unsustainable response to the phenomenon of chronic disease prevalence [6–8]. Globally, numerous strategies have been implemented to reduce hospital utilisation with variable success [6, 7, 9, 10], while overall, hospital demand continues to follow an upward trend [11]. If for reasons of economic and resource constraint alone, therefore, healthcare providers must foster practices that optimise outcomes for patients with complex health states, irrespective of the care settings [12].

Given the above issues, value-based healthcare has become a central tenet of Australia’s healthcare funding framework, with value conceived as sustainable, high-quality, ‘best-practice’ care, focusing on illness prevention and self-management [13]. Value-based care for chronic conditions, however, necessitates an investment in patient-provider time to foster effective communication, mutual understanding, rapport building, and ‘boundaryless’ care integration [14, 15]. In contrast, hospitals have high volume, high output settings that gear activity towards fast-paced episodic care [14, 16, 17], making time a scarce resource [14]. It appears, therefore, that hospital settings are misaligned both with the nature of health conditions that continue to form a substantive component of hospital activity, and the ‘value’ assigned to such care.

Furthermore, in consequence of the contemporary healthcare landscape, health complexity has become a much-discussed topic [4, 5, 18]. Its frequency in discourse signals health professionals’ repeated exposure in clinical practice to complex health states [18]. However, under the circumstances of a pressurised health system, it is unclear the extent to which health professionals share a holistic appreciation of health and illness [18]. For instance, discourse involving the term ‘complex’ is known to sometimes have a stigmatising connotation [18, 19], with ‘complex’ substituting for ‘difficult’ [18] or ‘burdensome’ [20] in relation to the patient. This implies that health complexity is problematic to the health system, rather than the reverse. It is an attitude that Searle frames as having a ‘world to mind direction of fit’, to denote an expectation that phenomena should fit our models, and the response of incredulity when they do not [21]. Such a mindset towards complexity should raise concern, however, because perception inevitably shapes behaviour, and in the context of healthcare, clinical practice [22].

In contrast, an attuned apprehension of the term ‘complex’ attends to the dynamic, unpredictable, and interconnected nature of health states that increasingly are being understood in terms of complex systems dynamics [18, 23]. In a positive sense, a complex systems’ framing elaborates the biomedical conception of health [22–24]. Moreover, a shift in healthcare thinking that is informed by the complexity sciences is gaining traction [23–27]. Yet despite this emerging reframing of health and illness, and globally, a gradual reorganisation of care systems [28], health professionals continue to confront challenges to providing well-integrated and holistic care. Barriers include health system and funding fragmentation [22, 29], healthcare costs [30], organ and disease-centric care [31], and the marginalisation of patient cohorts, deemed ‘difficult’ or ‘burdensome’ [19, 32].

It is in this context of a demanding health care environment, and in the setting of acute care, that the authors studied the clinician experience of complex care. The study’s aim was to apprehend the embodied and situated experience and meaning of complex caregiving in hospital settings. Study questions addressed the nature of ‘being’ a complex care health professional and by extension, the relationship of clinician perception and action in clinical practice.

## Theoretical Framework

The conceptual framework of this study integrates two fields: Phenomenology and complexity theory. Phenomenology is the study of ‘unprejudiced’ conscious experience from the perspective of the first person [33]. ‘Unprejudiced’, in a phenomenological sense, implies that analysis of phenomena is untainted by preconception and theoretical knowledge [34]. While this goal is contested within the paradigm of phenomenology, with some framing consciousness as inherently contextual [35] and embodied [36], core to all approaches is an interest in the intentional nature of consciousness, and its directedness towards phenomena in a perpetual quest for meaning [37].

Complexity theory, on the other hand, has migrated into the social sciences from its origins in the so-called ‘hard’ sciences; mathematics, physics, computer sciences [38] and network theory [39, 40]. Its uptake in the social sciences has been informed by a phenomenological alignment of its concepts with systems behaviours in the social world [41]. A ‘general’ form of complexity theory’s concepts assists in making sense of social systems’ behaviours [41]. For example, complex systems’ behaviour manifests as ‘*emergence*’, involving numerous factors that, in various combinations, generate irreducible phenomena [41]. In turn, emergent phenomena have a *non-linear* quality; their formation and behaviours are context dependent and probabilistically determinant [42]. Overall, complex systems’ behaviour is akin to a ‘black box’ of which the inputs and outputs are knowable, but the combinatorial behaviours generating such transformations are less certain [43].

**Fig. 1:**
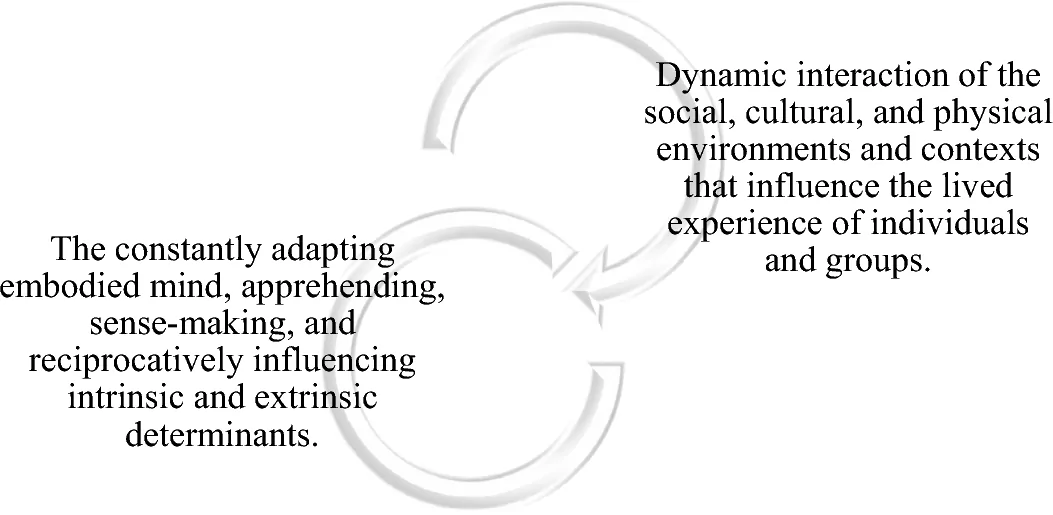
The systemic interaction of lifeworld—experience, perception, action, and context in concert [46, 47]

Seeing an alignment between complexity theory and phenomenology the authors developed a conceptual framework that they called complex adaptive phenomenology (CAP). As detailed in Fig. 1, CAP frames the embodied, conscious mind as continuously attuning apprehension and behaviour to an ever-changing lifeworld [36, 44]. In turn, lifeworld is shaped by, and generative of, the behaviours that are manifest within it [45]. The elements of lifeworld thus form an interactive niche; a reciprocal system of apprehension, behaviour, and manifestation [46]. This continuous interaction between mind, body, and the social and physical environment is generative of meaning [47] and informs CAP as the framework for this study.

## Methods

The authors’ overarching study involved clinicians from different disciplinary fields, working in one of three large metropolitan health services in Victoria. This paper explores the lived experience of the study’s medical participants. Ethical approval for the study was obtained from the lead health service’s ethics committee, each of the health services’ ethics and governance committees, and Monash University’s ethics and governance committee. Site-based principal investigators used purposeful sampling that sought variety in clinical experience so as to ‘confront difference’ [48]. This approach enabled the authors to develop an expansive horizon of complex care experiences. The medical participants consisted of two medical consultants (the most senior medical professionals) P1 and P2, one medical registrar (a highly experienced medical professional) P3, and one intern (junior medical professional) P4. All participants provided written informed consent to participate.

Data were collected using semi-structured interview, a common technique in phenomenological research [49, 50]. In the tradition of interpretive phenomenology, semi-structured interview questions entailed an openness to the interviewees’ lifeworlds, in an effort to capture participants’ most meaningful experiences [50]. Digitally recorded interview data were set down by professional transcription services and then sent to the relevant participant for comment. This was to ensure that the transcriptions offered a truthful account [51], and to foster a co-productive approach to inquiry [50]. Unfortunately, no responses were received, although this was unsurprising, given the busy nature of participants’ lifeworlds and the evolving COVID-19 pandemic that was engulfing healthcare services. The authors carefully read and systematically organised the transcriptions using NVIVO © software. As Van Manen suggests, the data were arranged according to emergent themes that evoked the meaning of ‘being’ a complex care clinician [52]. A condensed version of the themes is outlined in Table 1. The themes were then interpreted and described through the lens of CAP.

**Table 1:** Themes emergent from the interview data

Name	Description	References
Biomedicine	The biomedical model in relation to complex care	27
Care continuity, change and disruption	Barriers and enablers to continuity and their impacts	58
Communication and rapport	The function of rapport in complex healthcare	144
Fragmentation	Inter-sector fragmentation, funding fragmentation, and cultural isolationism	65
Complex patient attributes	Experiences that shape clinicians’ perspectives of a complex patient	62
The experience and meaning of complex care	Motivation to practice, responsibility for care, and the demands of the role	213

## Results

The authors selected the following detail from a rich suite of interview data to illustrate the unique nature of complex care. It is recognised, however, that the horizon of understanding is infinite, and the below offers but one interpretation [53].

### Biomedicine

Differing perspectives emerged in relation to how biomedicine informs complex healthcare. For instance, P1 viewed health conditions as problems, delineated according to those that could be “fixed” and those that could not. P1’s approach to problem solving emphasised guideline driven decision making. This was despite the known limitations of clinical guidelines that generally address clinical practice for single diseases rather than multimorbid states [31]. In contrast, P3 emphasised the limitations of clinical guidelines stating, “they’ll (patients) have three problems that are interacting with each other, so if you apply one guideline, you are, effectively, ignoring the other two problems.” Indeed, and not only would two problems be ignored, but so too would the emergent effects of all three problems in combination [41, 54]. P3’s methodology was that of piecing together parts of guidelines that best matched the unique circumstances of the individual patient, as follows: “So, … every patient gets a different formula, but it’s a formula based on… evidence, and you just pick the little bits that apply for that one person.”

Both P1 and P3 were committed to providing ‘best care’, the evidence for which is ostensibly captured in clinical guidelines that, in turn, are based upon the aggregated findings of randomised controlled trials (RCT) [55]. What remained unexplained however, was a method of assurance that the evidence cherry picked from each guideline constituted an optimal approach to individualised patient care.

The centrality of guideline driven practice caused the authors to reflect upon the nature of knowledge that informs complex care. They turned their attention to the matter of medical education that shapes how medical professionals come to frame health and illness [56–58]. The attitudes of emergent medical practitioners and researchers will inevitably influence the evolution of knowledge, and hence, clinical practice for complex care [31, 58–60]. After all, as Sturmberg and Miles observe, “the way we know defines the way we act” [22]. The authors thus pursued this line of inquiry with P4, the most junior of the study participants, by asking how their recently completed tertiary education informed their understanding of health complexity. Their response is below.

I think it’s mostly single disease-oriented education, but having said that though, I don’t necessarily think that the university course could have, or should have, done anything different. Because there are just some things that you pick up with experience… the uni experience doesn’t encapsulate the whole patient thing well, but I also don’t really necessarily feel like they could do that much better.

While P4 did not view their university curriculum to be deficient, they simultaneously did not expect to attain clinical expertise in under twenty years of practice, an expectation that had been normalised by their medical supervisor. This extensive time-frame contrasts with the rapid and continued progression of disease specific research that informs the development of ‘evidence-based’ clinical guidelines [31, 59]. The possibility of an alternative research paradigm to augment the practice of complex healthcare was not considered by any of the participants. RCT as ‘best evidence’ was thus ‘baked in’ to their thinking.

### Care Continuity

Care continuity may be conceived as entailing three key elements: Continuity of relationship, information, and management or coordination [28, 61]. The participants varied in how they valued and exercised relational continuity. P2 experienced joy in witnessing patients’ engagement in life with, and beyond, their chronic illnesses. Enduring relationships were thus central to P2’s experience of professional fulfillment, providing tangible validation of their care. They explain,

It’s a journey, and I often see these patients in their adolescent years…and, you know, you get to know them through high school, university, and families. And its lovely to see when they first start – we have weddings, and we have marriages and it’s that life journey of a patient in that outpatient setting.

However, P2 struggled with the disconnect experienced by older, multimorbid, patients in the inpatient setting. While these patients may have had long-standing relationships with disease specialists in the community, this connection did not translate to the hospital environment. P2 explained the impact of this disjuncture on families and patients as follows: “I think it’s one of the biggest complaints [from families and patients] when you’re doing the ward round… And I still struggle with about how could we, as humans, refuse to see somebody.”

P1, on the other hand, saw relational continuity as assurance that any health gains made in the inpatient setting would not unravel upon hospital discharge. P1 felt accountable for the expense of hospital-based care and, as widely acknowledged in the literature, the need to reduce resource wastage [62]. P1 reasoned, “We’re an expensive business and we put a lot of resource into someone”. Acutely aware also, of health system fragmentation [63, 64], P1 stressed the importance of timely medical follow up to protect against any erosion of this investment of resources. They explained, “…when they [patients] leave the service, they can feel a bit lost. It’s just like the rope’s been cut”. P1’s reflections were consistent with Ljungholm et al. [28] who found that hospital discharge posed a point of clinical bifurcation with an associated heightened risk of disconnection between patients and clinicians. Furthermore, like P2, P1 noted a discontinuity of care within the inpatient setting, explaining, “…you can have five days and five different doctors in the hospital”.

P3, on the other hand, saw clinician-patient continuity in terms of having the same clinical ‘team’ oversee care, even though various doctors might attend to the patient over the course of their hospital stay. P3’s continuity was thus more managerial and informational in nature, with continuity construed as consistency in senior doctor oversight of the clinical team. P4 however, noted that patient choice was also a factor in care continuity. They explained they rarely discharged a patient without offering support services but not all patients were accepting of these. P4 felt that by declining ongoing services patients placed themselves at a greater risk for re-presenting to the hospital.

Differing perceptions of what constituted care continuity, including time, place, relationship, coordination, and information, thus influenced how the participants perceived continuity. P2 viewed continuity in terms of enduring clinician-patient relationships, P1 as having a relational presence across transitions of care, and P3 as coordinating informational consistency within the care team, irrespective of the fast-paced, time scale of the inpatient setting. The success of care continuity was also patient dependent however, irrespective of the clinician’s preferred approach.

Finally, just as patient care continuity was considered important, so too was continuity within the clinical team. P1 referenced the importance of a “stable workforce” because, “it costs a fortune when you’ve got an unstable workforce …Just the fragmentation in the actual efficiency of the service”. The literature supports P1’s assertions, since a stable workforce fosters the development of collaborative team learning and evolves to become a ‘dynamic capability’ within the group [65]. Dynamic capability manifests as tacit, intersubjective,and experiential knowledge that generates efficient and innovative ways of working in and across teams [66].

### Communication

Effective communication is essential to the smooth functioning of a health system and safe, effective patient care [67]. It is critical to clinical information exchange across professions, teams, organisations, patients, and carers [28], and fundamental to sense-making one’s dynamic experience of lifeworld [67]. A multitude of communication forms exist within health services including digital technologies [62], email, videoconference [68], telehealth [69], and the electronic medical record [70], and participants had mixed experiences in this regard, both in relation to patient care and within the healthcare sector broadly.

For instance, P1 found that outpatient appointment letters took an excessively long time to be processed and to reach the patient in the community setting, sometimes causing patients to miss their appointment. Furthermore, the letters were printed in English and thus failed as a communication tool for people of non-English speaking backgrounds. On top of this, P1 explained that the letters’ way-finding instructions were confusing, so that patients easily became lost trying to find their clinic location within the hospital setting.

On the other hand, P1 found simple technologies, such as email, to be a highly effective tool in managing patient care, as follows.

…if you’ve got an engaged and caring daughter who is holding up the mother with heart failure and their daily weighs and blood pressures and all that, … I do get reports from the daughter …And you find it’s good to receive these…it strengthens relationship, and they take on the feedback, and actually these people do well... So, I spend a fair bit of time doing that [emailing carers].

Effective communication, incorporating simple technology, was seen to strengthen the clinician-patient relationship and enhance the experiences of both. A strong therapeutic relationship coupled with ease of access functioned as a ‘clinician-patient-carer’ niche that enabled P1 to offer a dynamic plan of care, continuously adjusted to the patient’s status.

Similarly, P2 felt it important to be accessible to their patients. Clinic nurses facilitated this expectation by transferring patient phone calls from the community directly to P2’s mobile phone. P2 explained the importance that they placed on ease of access, recounting a time when a clinician incorrectly advised one of P2’s patients that they were not available. Following this incident, the patient had expressed their disappointment to P2 as they had fully expected to be able to speak with them. In response, P2 pressed that they had been available, and that the clinician had misinformed the patient. P2 then followed up with that clinician to explain the impact of their miscommunication, which the patient had experienced as a breach of patient-clinician trust. In P2’s view this had potential to undermine patient engagement and thus patient outcomes.

However, there were also clear differences in what participants valued as communication. For example, P4 was less interested in relational communication, and as they put it, “chit-chatting about their dog”. Instead, they were focused on extracting patient information by which to build a diagnostic and prognostic picture. This difference in valuing may reflect the participants’ contrasting levels of experience. Clinical rapport, while important to P1 and P2, was not front of mind for P4. However, the time available for relational care appeared also to be influential. P4 described being focused on “getting the job done”, “identifying what the patient thinks the issues are” and using their time to obtain feedback on the patient’s progress. Their framing of clinical work in the hospital setting resonated with Foucault’s ‘clinical gaze’; focused on the task at hand, it was methodical, and distanced [71].

### Fragmentation

The theme of fragmentation permeated the lifeworld of participants and their accounts of complex healthcare. Inter-sector funding disparity, differing clinician schedules, and the resistance of health professionals to work outside of their usual realm of operations, contributed to this division as P2 explains.

We now have these services [for example, case conferences] where we have …some of these patients attending, and that’s basically to get all the specialty teams together to talk about the complex management of the patients, which they should be doing with the GPs but we’re not flexible enough… we’re in a hub and we don’t then go outside that hub. And then so how do the GPs get a family meeting with the cardiologists, neurologists, and renal physician all at the same time? It’s nigh impossible.

In consequence of the above constraints, care that could be provided by primary care providers may be set aside, such as advance care planning conversations, that are ideally held with the patient’s long-standing provider [72]. The hospital physician, with only limited exposure to the patient’s life journey, must then take up the mantel, as P2 explains,

…where do the GPs sit with the role of end-of-life discussions, and GPs are pushed for time. They’ve got financial constraints. There’s no rebates really for these things, so it’s often not done…So I think that some of these situations we’re dealing with in hospital acutely is because we’re not doing it in the community.

Fragmentation of care also presented at the point of hospital discharge as noted above by both P1 and P4. Hospital discharge posed a point of divergence where the health improvements made as an inpatient could either be sustained or come undone. P4 had conceded that ‘most patients don’t cope well’, while P1 laboured to ensure patients were followed up in a timely manner following discharge. Both accepted however that the world outside of the hospital walls posed challenges to the efforts of their care over which they had minimal control. In myriad ways, fragmentation had a great presence in the lifeworld of participants, and that of their patients whose care journeys intersect community and hospital care settings.

Yet, while care fragmentation posed a threat to patient outcomes, some of the participants conveyed that hospitals were extending themselves too far in the community-based care space. That really, community-based care was the realm of general practice, while that of the hospital was acute care. P1 explains,

…perhaps they [patients] should be using the local resources, including GPs etc, and maybe going to the GPs first and the GPs should be able to contact us with questions like tweaking medications, or refining things. But that doesn’t seem to happen and the reason for that is we provide a more reactive service.

P1 thus felt that perhaps GPs were less engaged in managing the undulating clinical states of patients because hospitals have developed models of care that out-reach into the community, providing a reactive and supportive clinical response [73]. However, the hospital based outreach models to which P1 referred were implemented precisely because the substantive primary care response had been unable to curb an unsustainable rise in ‘avoidable’ hospital attendances [74].

### Complex Patient Attributes

The participants’ experience of health complexity aligned with the literature, presenting as a combination of clinical, social, and behavioural attributes [18]. P2 cared for a diverse range of complex conditions across both young and older demographics. Many patients had potentially life-limiting conditions, which made dealing with death across the lifespan a fundamental aspect of P2’s practice. This emotionally weighty aspect of their role was made more difficult in the context of family complications. P2 described the difficulty of breaking bad news to family members that, for whatever reason, choose to not communicate with one another.

P2: …so how do we protect our clinicians in the hospital …. from that emotional burden of telling somebody that they’ve got terminal cancer to five different people, and the same story, and by the time you get to the fifth time, it’s kind of like you’re kind of emotionally drained where it’s not an empathetic communication.

In addition, then, to dealing with the multitude of physiological and social complexities that are inherent to complex care [18], the participants often engaged in challenging conversations with patients and their relatives. Unsurprisingly, the taxing nature of this work might leave one feeling depleted. For instance, P1 expressed, “I just feel sometimes that a lot of these patients really suck the life and energy out of our …team that are doing their best, and perhaps they should be using the local resources…”.

Furthermore, organising complex care regularly necessitates an assessment of carers’ capacity, given their crucial role in supporting the patient in the community setting [28]. Yet often, when patients are elderly, so too are their carers, and this adds to the complexity of effecting an achievable discharge plan of care. P2 highlighted that assessment of the patient’s social supports may reveal neither is able to safely care for the other. In such instances a series of actions ensue, including legal proceedings to secure guardianship over decisions concerning the patient’s ongoing clinical needs and functional supports. In consequence however, the patient whose acute medical issues have resolved, may languish in a hospital bed until an appropriate discharge plan can be enacted. P2 expressed frustration at such scenarios, which they felt could have been predicted and managed proactively in the primary care setting, to mitigate a hospital-based family “crisis”.

Finally, so-called ‘complex patients’ [18] tend to have numerous health conditions that, as P1 explained, always entail “…a fair bit of follow up” and “…there’s always homework to do after the clinics”. According to P1, some patients, “…never get discharged”. As P3 explained, unless they were children presenting to the emergency department, or women receiving maternity care, patients within the hospital setting generally had chronic conditions, irrespective of their reason(s) for admission. Furthermore, complex health states required the continuous adjustment of treatment. In P3’s words, “They’re not sort of plug and go, and yeah, things crop up all the time”.

### The Experience and Meaning of Complex Care

Overall, it was making a difference to people’s lives that motivated the practice of all four participants. From the many examples within the data two have been selected that reflect the deep-seated dedication to patient care that was common to all. P2 expressed, “… it’s a whole myriad of things about helping people and then when you actually make a difference, seeing how you have made a difference and improved the quality of life.”

Similarly, P4’s motivation to pursue a career in endocrinology was that chronic conditions care can often lead to lasting clinician-patient relationships. Aside from the fact that “the numbers don’t lie”, they believed this speciality fostered “… longitudinal work, so you are able to develop a long-term relationship with people and get more of that long term trust and so that you are able to do more, you know, effective advocacy and campaigning for your patient.”

Yet opportunity to reflect on the meaning of their work was constrained by the pressure and pace of work in the acute care setting, as P1 explains.

I think people [clinicians] sort of come in and just get on that treadmill that’s in one direction, and complete it, get off at the end of the day and get back on the next day. And … I don’t know if there’s an opportunity – there are many opportunities, of if people really think about things and try to...reflect on them.

In this vein P4 highlighted and normalised the non-stop nature of their work. A typical workday might commence at 8 am in the morning and finish at 9.30 pm. P4 explained, “but then you get in early and finish late a lot of the time as well”. However, these hours represented a light workload in comparison to P4’s surgical rotations, where working hours were from 6.30 am through to 10.30 pm, and then back again the following day. When asked how they coped with these grueling schedules they replied, “…it’s just like—I mean, everyone does it, so you just rock up and you just do the job and get it done, and then go home.”

## Discussion

The lifeworld experiences of the study participants were contingent upon myriad shifting elements both within and external to the hospital. Most prominently, these clinicians, specialising in the dynamic interrelatedness of health and social issues, were ensconced in a system that is geared toward acute, episodic care [69]. Yet, episodic healthcare is a poor fit for the patient with chronic and complex health needs [75] and by inference, the practical needs of their clinicians.

Foundational to the episodic system of hospital care is the biomedical model of health and illness, a clinical approach that reduces disease to the cellular level [17, 60, 76]. So entrenched was this model in the lifeworld of participants that the possibility of reframing hospital care to better match the complex and integrated needs of more than 50% of its patients barely surfaced [5]. Instead, participants expressed that the world external to the hospital, particularly primary care, should, or could, adjust to better match the needs of patients and lessen the burden of complex care within the hospital environment.

Moreover, with biomedicine underpinning medical care, medical practice remains largely disease centric [31, 58] and therefore, irrespective of where care is provided, healthcare is itself fragmented at the most basic level. Even despite their predominantly multimorbid patient cohort, participants viewed disease centric clinical guidelines as ‘gold standard’ [22]. Other ways of ‘knowing’ were considered less acceptable as they would violate the rule of invariance inherent to the RCTs underlying clinical guidelines [22]. Yet despite this, other ways of knowing *were* being implemented. The physicians wove together pieces of guidelines in accordance with the patients’ conditions, and this kind of knowledge was deemed acceptable because it still involved the use of guidelines. It is, however, an inherently experimental approach, wherein each patient is, in effect, a clinical trial [31]. It is furthermore an unfortunate truth that such experimentation frequently produces adverse effects that impair the patient’s function and well-being [77]. A fundamental inequity in the focus of biomedicine is thus revealed in its evidence base, which underscores the model’s limitations also [31]. Given the ubiquity of multimorbidity the need for fit for purpose research methods for chronic and complex disease management is manifest. However, even the famous Chronic Disease Model refers to the use of evidence based guidelines [78] that ignore the difficult issue of multimorbidity [3, 79, 80].

The interwoven and enduring nature of chronic and complex conditions further differentiates complex care by requiring clinicians to extend care beyond the walls of the hospital, into social and relational realms [19, 28, 63]. This work appears to contrast with specialist care that, according to P2, effectively differentiates the external environment from the hospital setting. The physicians, especially those most clinically senior, instead worked within person-centred niches of care that, inclusive of significant others, entailed ongoing clinician-patient connection within clinics, via email, or by telephone. P1 for example, integrated care across the traditional acute and community divide [81], by establishing trusted relationships that fostered carer capacity building for supported self-management. Interconnectedness beyond the hospital enabled rapid responsiveness to the patient’s changing care needs, and thus was generated an ‘autopoietic’ relational and self-sustaining care system [46].

According to Maturana et al. [46], social niches, such as that mentioned above, provide the indispensable social architecture enabling individuals to survive and thrive. This kind of architecture seems fitting for the dynamic nature of chronic and complex conditions that, in effect, behave as complex systems [54]. While episodic care models assume health issues to resolve, chronic and complex conditions are instead enduring and evolutionary [4]. Moreover, chronic conditions fluctuate unpredictably between states of stability and instability [82]. The dynamism of chronic disease makes clinician familiarity with the patient all the more important as knowing the patient’s ‘normal’ status, and having an understanding of their personal values, life history, and clinical evolution, assists in personalised clinical decision making [28].

Yet the time for relational care is scarce within the acute care setting. Laitinen et al. [14] stress that time, a critical element of effective healthcare, too often is truncated in episodes of care. In consequence, patients “struggle to survive” an “anachronic” and fragmented health system [15], just as clinicians grapple with the pressure and pace of their lifeworlds and the hospital systems’ ever present focus on ‘progression of care’ [83]. Healthcare time, as an intervention in its own right, thus appears to be both undervalued and overpriced, at the expense of patient care and clinician experience [5]. Moreover, time pressure and fatigue have the effect of constraining clinicians’ capacity for critically reflective practice [14].

Still, and despite the frustration caused by the above constraints, or perhaps because of these, the participants normalised many of their lifeworld experiences. Husserl describes such normalisation as the ‘natural attitude’; an unreflective ‘every-day’ demeanour that constitutes one’s general engagement with lifeworld [34]. A natural attitude appeared to shape participants’ assumptions about the role of the hospital, general practice, the patient, the health system, notions of healthcare, and more. While there is much to question about present systems of care for complex health conditions, as P1 explains, clinicians tend to get on the treadmill of work and by the time they alight, the thirst for reflection may well have dissipated. Yet intuitively and readily, the participants described their unique practice in intricate and interwoven detail. Theirs was not simply disease management; rather, it was a form of practice individually tailored to the patient’s physical predicament and their contexts of living. The capacity for this dynamic and responsive practice to effect change in a patient’s health status, or to at least allow patients to engage in a life additional to disease, instilled great meaning for this work.

That said, healthcare “is an expensive business” as P1 points out, and the complex health states contributing to that expense remain on the rise [75]. Reflection upon practice, models of care, and perceptions of health and illness, is thus critical to addressing conceptual barriers to what is best for complex care [22]. Often, hospitals are framed as being the wrong place for chronic conditions care, especially for the elderly [84], coupled with the assertion that chronic disease management should be provided elsewhere [7, 85]. Yet, as chronic conditions affect more than half the population [5], hospitals must inevitably come to better reflect community needs [15]. This requires the adoption of emergent and innovative ways of apprehending health and illness that extend beyond the single disease-centric thinking traditionally informing clinical practice and models of care [23, 57, 58]. In turn, such a shift would require broad awareness of the natural attitudes’ effect upon framing and decision making in relation to health, illness, and healthcare policy generally. Such apprehension opens the door to intentional engagement with the unique nature of complex care practice and the horizon of its inherently interwoven architecture [63, 86].

In contrast, the effect of simplifying the complex is for the complex to behave as complex systems do. That is, to resist containment [87] and continue to fuel the already ballooning problem of hospital and health system demand [75]. Put differently, to be confined to an epistemology that insists upon a ‘world to mind direction of fit’ [21], is to be enmeshed in what complexity science terms an ‘attractor’ [41]. Attractors are state spaces made up of coherent elements that dominate the overall behaviour of a system [41]. Episodic, biomedical care acts as an attractor within acute care settings [14, 17]; it dominates and coheres thinking which, in turn, enforces adherence to forms of health professional research, education, and practice [22]. Consequently, these deliver what Chan et al. [63] assert to be a mismatched system for the increasingly complex needs of patients. The experiences of physicians whose practice is that of complex healthcare lends support to their assertion.

One might thus ask how acute care for chronic and complex conditions might be enhanced. To this open question the authors suggest environmental attunement to the needs of the complex patient and by extension, the needs of clinicians that provide their care. In this regard the authors refer not just to hospital doctors, but to primary care providers, specialists, and community practitioners also. In view of the fact the complex health issues follow patients and their carers across care settings, structured collaborative approaches that bridge healthcare sectors may prove invaluable both to providers and patients. Indeed, Pearl and Madvig articulate how Kaiser Permanente demonstrated clinical and financial success through such an approach [88]. Collaboration functions as a ‘niche’ [46] wherein providers are connected in a relationship of care, each with their jurisdiction and role, but all in collaborative support of the patient, rather than the patient having to negotiate what can appear to be divided worlds [88].

Furthermore, the authors advocate for the progression of a research paradigm that recognises multimorbidity, not as a set of single diseases, but as complexes that are worthy of their own ‘gold standard’ guidelines and interventions. Multimorbidity needs augmentation of emergent, fit-for-purpose, clinical research efforts to optimise this field of practice [77, 89, 90]. In this respect, the advent of machine learning (ML) from so-called ‘big data’ may assist in strengthening the knowledge base for complex care [77]. ML, also called artificial intelligence, offers an evolution of biomedicine by shifting this established field into the realm of complexity [77]; a conceptual domain that many argue is better suited to complex care [22, 23, 54]. In addition, cluster analyses of diseases, health service utilisation, and treatment burden, such as that undertaken by Juul-Larsen et al. [90] offer an integrated, multi-system interpretation of health complexity to help guide the development of models of care, clinical pathways, and tailored healthcare networks, (niches) [46]. Similarly, the emergent field of syndemics investigates complex health conditions as clusters that are inclusive of comorbidity and psychosocial determinants [86, 91]. Finally, Harrison et al. [89] note the benefits of an organ centric approach to disease clustering and management, although this aligns with restricted, more than inherent, complexity [41]. Emergent research techniques by which to examine the nature of multimorbidity, such as those mentioned above, portend the development of innovative practices, and thus warrant considerable focus in healthcare policy and research.

The issue of time also requires thought. Hospitals demand a pace of work that constrains opportunity for relational care and clinician reflection [14], and yet these are fundamental to good healthcare [82, 86, 92]. Time is a key tool in both clinical and reflective practice and must therefore be better perceived and valued as such [28, 82, 92]. Hörburg et al. [93] explain, reflection sensitises one to the complexity of lifeworld experiences. While sense-making of phenomena is a continuous process [94], the rich understandings of deep reflection exceed those of in-the-moment insights [50]. Such reflection might attend to the social system that is constitutive of healthcare.

Structures, like healthcare, while emergent of discourse, also bear influence over perception and action [21, 95]. Discourse is both agentic in, and constrained by, the power within social structures [21, 95]. This reciprocal relationship shapes the normative values informing healthcare [76]. Time for reflection allows for a continuous critique of the beliefs and values underpinning this discourse [93, 96]. Yet the hospital environment associates time with tasks [14] and the normalisation of this constraint enables a continued dominance of the substantive discourse. Clinician reflection and influence over the system shaping their lifeworld is thus constrained by its time-limiting structure [97]. To correct this imbalance, the authors recommend that time for reflective practice be incorporated within models of acute care workforce, such that the assumptions informing healthcare lifeworld may be illuminated, critiqued, and potentially changed.

Finally, health professional education and training might come to better reflect how chronic conditions present in actuality; not as confined to discrete systems [58], but as complex systems within complex entities that live in a complex social world [23]. In short, the authors propose that complex care has its own ground, to be valued for what it is; not as a problem to be solved, but as a paradigm of care like no other. Hence, the medicine engaged in this care, more than ‘general’ [3], is ‘complex’; a term that arguably better reflects the science holding much promise for this field [27].

## Limitations of this Study

This study entailed just a small group of four participants and thus cannot be considered representative of the wider community of physicians whose work entails complex care. However, phenomenology does not attempt to produce generalisable outcomes. Rather, the aim of phenomenologically oriented research is to unveil that which lies hidden in plain view, to unearth normatively held assumptions, and to elicit reflection upon these [52]. In addition, the authors would ideally have had feedback from participants about their transcripts, but the busy nature of health care coupled with the disruption of the COVID-19 pandemic made this difficult to achieve. In an effort to minimise bias, the authors exercised what Dahlberg and Dahlberg term ‘bridling’, which is to reflexively exercise restraint, by pulling back from interpretations and interrogating own assumptions, prior to committing thought to paper [98]. Lastly, while interview questions were framed to illicit the nature of ‘being’ a complex care medical doctor, this focus may simultaneously have led participants to attend less to other lifeworld elements, such as the interdisciplinarity that is common to complex care models [12, 63, 84, 99]. The authors advise, however, that the interdisciplinary nature of complex care is included in the overarching study. Another author might interpret our data in a different light, while the same authors might also, at another point in time. Hence, this study is but one account that is emergent of the horizon of the complex care physician’s lifeworld.

## Conclusion

The experiences of this study’s physicians offer rich insights into how the ubiquitous phenomenon of health complexity is apprehended and addressed in the hospital setting. Through a complexity informed phenomenological lens, this study revealed complex healthcare to be a rewarding but also demanding field of clinical practice. Complex care is meaningful work that can generate positive change in the lives of people with chronic and complex health conditions. However, it is also challenging work, made more difficult by the system in which it is situated; a system that is geared to fast-paced, single disease oriented care [17]. The authors contend that complex healthcare must delineate its unique ground for this practice to be optimised. Such delineation requires a rethink about the nature of health and illness in the person in their lifeworld [77]. This paper has offered suggestions as to what such a rethink might entail, but these by no means complete the picture. That is because health is a complex adaptive, constantly evolving, and dynamic system [54]. There can, therefore, be no end to the development of the complex care paradigm. However, continued intentional apprehension of this speciality’s unique and evolving horizon stands to benefit the course of its trajectory and impact.

## Data Availability

The datasets generated during and/or analysed during the current study are available from the corresponding author on reasonable request.
